# DESH negative normal pressure hydrocephalus: can patients still benefit from shunt insertion?

**DOI:** 10.1186/2045-8118-12-S1-O52

**Published:** 2015-09-18

**Authors:** Tarek Mostafa, Claudia Craven, Neekhil A Patel, Edward W Dyson, Samir A Matloob, Aswin Chari, Patricia Haylock-Vize, Simon D Thompson, Syed N Shah, Andrew R Stevens, Huan Wee Chan, Jinendra Ekanayake, Ahmed K Toma, Laurence D Watkins

**Affiliations:** 1 Victor Horsley Department of Neurosurgery, National Hospital for Neurology and Neurosurgery, Queen Square, London, UK

## Introduction

Selecting probable idiopathic normal pressure hydrocephalus (INPH) patients for shunt insertion presents a challenge because of coexisting comorbidities and other conditions that could mimic NPH. The characteristic appearance of DESH (Disproportionately Enlarged Subarachnoid Space Hydrocephalus) on brain imaging has been shown to have a high positive predictive value in identifying shunt responsive INPH patients (SINPHONI trial). However, the negative predictive value of this radiological sign was not clearly demonstrated.

## Methods

A single centre retrospective study of probable INPH patients, who underwent ventriculoperitoneal shunt insertion. Case notes were reviewed for clinical presentation, prognostic tests used and postoperative improvement. Shunt responsive INPH patients were identified as those having objective improvement in their walking speed, neuropsychological assessment as well as subjective improvements in gait, continence and memory, one year post operatively. Preoperative images were reviewed for DESH sign (2 independent reviewers). Negative and Positive Predictive Values (NPV and PPV) of DESH sign were determined post analysis.

## Results

A total of 103 probable INPH patients were included (31 were DESH positive (30%) and 72 were DESH negative (70%)). A total of 78 patients showed measurable improvement 1 year post shunt insertion (76%); 24 (31%) of these patients were DESH positive and 54 (69%) were DESH negative (P = <0.001). Therefore, the DESH sign had a PPV of 77% and a NPV of 25%.

## Conclusion

In our data, the presence of DESH sign demonstrates a high positive predictive value of 77%, in agreement with SINPHONI trial data. However, it has shown a low negative predictive value. We conclude that probable NPH patients should not be excluded from having shunt insertion based on the presence of DESH sign alone.

## Abbreviations

DESH: Disproportionately Enlarged Subarachnoid Space Hydrocephalus; NPH: Normal Pressure Hydrocephalus.

